# Identification of endophytes with biocontrol potential from *Ziziphus jujuba* and its inhibition effects on *Alternaria alternata*, the pathogen of jujube shrunken-fruit disease

**DOI:** 10.1371/journal.pone.0199466

**Published:** 2018-06-26

**Authors:** Jian-Feng Lang, Xue-Liang Tian, Ming-Wang Shi, Long-Xian Ran

**Affiliations:** 1 College of Forestry, Hebei Agricultural University, Baoding, Hebei, People’s Republic of China; 2 Department of Plant Protection, Henan Institute of Science and Technology, Xinxiang, Henan, People’s Republic of China; 3 Hebei Key Lab of Forest Germplasm Resources and Protection, Baoding, Hebei, People’s Republic of China; Universita degli Studi di Pisa, ITALY

## Abstract

Endophytic strains were isolated from different parts of a healthy “Dongzao” jujube (*Ziziphus jujuba* Mill. ‘Dongzao’) to find biocontrol agents against jujube shrunken-fruit disease caused by *Alternaria alternata*. The strains were screened using *A*. *alternata* strain CN193 as the target pathogen. The nutrient competition for all isolates was studied using the dual culture, and their inhibitive capability was tested by measuring the inhibition width of filter paper disks with filtrate. Influence of filtrate from the selected strains with strong inhibition of mycelial growth on spore germination was studied with hanging drop method on concavity slides. Colonization in the jujube leaves was assayed using a rifampicin-resistant mutant of strain St-zn-34 as the screening marker. Strains were identified based on their morphological, physiological, and biochemical characteristics, 16S rDNA sequencing, and phylogenetic analysis. A total of 81 endophytic strains were isolated from the stems, leaves, flowers, and fruits of winter jujube. Among these isolates, 14 strains showed strong antagonism against *A*. *alternata*. Further study showed that the filtrate of strains St-zn-9 and St-zn-34 could inhibit the mycelial growth of *A*. *alternata*, and the widths of their inhibition zone reached 6.14±0.03 mm and 8.27±0.09 mm, respectively. However, strain St-zn-34 showed stronger inhibition on spore germination than strain St-zn-9. St-zn-34 could significantly reduce the spore germination rate of *A*. *alternata*, and the spore did not germinate at all or the germ tube was very short. A rifampicin resistant-derivative of wild-type strain St-zn-34, which was designated as St-zn-34^r^, was obtained by transferring the strains to media with stepwise-increased rifampicin. Colonization assays indicated that St-zn-34^r^ could colonize in jujube leaves, and the population of St-zn-34^r^ was 1.2×10^3^ CFU/g FW after inoculation for 30 days. Except for its salt tolerance, St-zn-34 was the closest to those of *Bacillus subtilis*. Thus, the strain was identified as *B*. *subtilis*.

## Introduction

Jujube tree is a unique fruit tree species in China. The planting area and output for jujube in China account for 95% of the world’s planting area and output for this fruit. However, jujube shrunken-fruit disease (JSFD) is prevalent in jujube plantations in China [1–4]. This disease influences jujube yield and quality and results in total crop failure. Numerous studies have been conducted to prevent the shrunken fruit disease. Chemical control is the most common method applied to remedy this disease [5,6]. However, the repeated application of chemical pesticides throughout the growth period of jujube hardly improves the condition of the jujube plant but rather results in damages to ecological balance, high pesticide residue, and drug resistance of pathogens. Exploring a new environment-friendly and effective biological pesticide to reduce these negative effects is currently a research hotspot to decrease the use or even replace chemical pesticides. Endophytes are microorganisms in healthy plant tissues which do not evidently harm plants. These microorganisms could be isolated from plant tissues through surface sterilization. Endophytes possess high colonization ability in the host [7] and provide beneficial effect to the host [8,9]. Many studies have proved that the endophyte metabolite could kill pests and bacterial activity [10,11]. Therefore, exploring and using strains with active substances against disease and pests in plant endophyte can be used as biological agents to prevent shrunken fruit disease of Chinese jujube. Existing studies on the selection of associated antagonistic bacteria have mainly focused on rhizosphere soil bacteria [12], jujube endophytic bacteria [13,14], and jujube leaves [15]. Isolation of endophytic fungi, bacteria, and actinomycetes from different parts of healthy jujube to select antagonistic bacteria against the pathogen of JSFD has not been reported previously.

In this experiment, endophytic fungi, bacteria, and actinomycetes were isolated from different tissues of healthy jujube. Strains with inhibited pathogen activity of JSFD were selected by testing their nutrition competition, production of antibiotics, and inhibition of pathogenic spore germination. The colonization capacity of these endophytes on jujube trees was tested. Additionally, strain was identified through morphological observation, physiological and biochemical reaction, 16S rDNA sequencing, and phylogenetic analysis. This study was performed to screen endophytic strains with antagonistic activities against the pathogen of JSFD, and lay a foundation for a potential effective biocontrol agent against JSFD.

## Materials and methods

### Plant materials

Samples included fresh branches, leaves, flowers, and fruits of “Zhanhua Dongzao” jujube. The samples, which included mature and tender tissues and were collected using a pair of scissors, were obtained from the Xinxiang Forest Seedling Cultivation Demonstration Base on July–October, 2014. Samples were stored in a refrigerator at 4 °C and isolated after 24 h. The samples which could not be processed in a short time were stored in a refrigerator at 4 °C but were isolated within 48 h.

### Pathogen

*Alternaria alternata* CN193 strain used in the experiment as the pathogen of JSFD was originally isolated from the jujube fruits with typical symptom of JSFD in jujube plantations of Tangxian County, Hebei Province, and provided by the Forest Pathology Laboratory of Hebei Agricultural University, P. R. China[16].

### Culture media

Potato dextrose agar (PDA) (in g/L: Potato 200, Dextrose 20, Agar 20 and 1 L distilled water) and beef extract-peptone media (in g/L: Beef extract 5, Peptone 10, NaCl 5, Agar 15 and 1 L distilled water, adjust the pH to 7.4 with 1 N NaOH) were used to isolate and purify the endophytic fungi and bacteria, respectively [17]. Fermentation cultures for the fungi and bacteria were based on Potato dextrose (PD) (in g/L: Potato 200, Dextrose 20 and 1 L distilled water) and Luria-Bertani (LB) media (in g/L: Tryptone 10, Yeast extract 5, NaCl 10 and 1 L distilled water, adjust the pH to 7.4 with 1 N NaOH). The endophytic actinomycetes were isolated and purified using Gauze’s medium No.1 (in g/L: Soluble starch 20, KNO_3_ 1, K_2_HPO_4_ 0.5, MgSO_4_^.^7H_2_O 0.5, NaCl 0.5, FeSO_4_^.^7H_2_O 0.01, Agar 20, and 1 L distilled water), and the actinomycete fermentation culture used the Millet medium (in g/L: Millet 20, CaCO_3_ 1, KNO_3_ 1, K_2_HPO_4_ 0.5, MgSO_4_^.^7H_2_O 0.5, NaCl 0.5, FeSO_4_^.^7H_2_O 0.01 and 1 L distilled water) [18].

### Isolation method of jujube endophyte

Endophytic fungi were isolated by tissue culture method [19]. The samples were soaked in 75% alcohol for 3–5 min, followed by 3–4 times rinsing with sterile distilled water. Then the samples were surface sterilized with 0.1% (w/v) HgCl_2_ for 10–30 s, and washed with sterile distilled water for 5 times. After disinfection, the plant tissue was cut into 0.5–1.0 cm slices on the laminar flow cabinet with sterile blades. Then the small slices were placed on the PDA medium plate containing 50 mg/L streptomycin, and cultured in the dark for 7–21 days at 24–26 °C.

The endophytic bacteria and actinomycetes were isolated by tissue homogenate culture method [20,21]. The samples were soaked in 70% alcohol for 5min, sterilized with 0.9% bleach powder for 20 min, submerged in 10% aseptic sodium bicarbonate solution for 15 min, and finally washed with sterile water for 5 times. About Half gram of the sterilized samples were placed into a mortar with 2 mL sterile water, and fully ground. One hundred microliter homogenate was applied with a triangular glass rod onto LB and Gause’s medium No.1 plate, respectively, and then cultured in the dark for 5–14 d at 27–29 °C. The last washing fluid was transferred onto all isolation culture media and cultured for 30 days at 28 °C for observation of sterilization effect. The purified strains were stored on the PDA slope at 4 °C for short-term preservation, while 20% glycerol was used as antifreezing agent for long-term preservation of all strains at −80 °C.

### Preliminary screening of biocontrol strains

Biocontrol strains were screened preliminarily through dual culture [22]. Specifically, the endophyte was inoculated at one end of the PDA plate (d = 90 mm), while the pathogen of JSFD were inoculated on the other end of the same plate at a distance of approximately 3 cm. The plate was cultured as inverted at 28 °C. Colony growth and inhibition of pathogenic bacteria were observed successively daily for 2–7 days. At the same time, the plate inoculated only with pathogen of JSFD was considered as the control group. Three replicates of each treatment were used, and the screening tests were repeated two times. Colony diameters of the tests and control were measured and recorded. The inhibition rate was calculated as follows: inhibition rate (%) = [(colony diameter of the control group—colony diameter of the test group)/ colony diameter of the control group]×100%.

### Inhibition effect of fermentation filtrate on the mycelial growth of *A*.*alternata*

The culture media inoculated with endophytes were placed in a constant-temperature oscillation incubator for the fermentation culture of fungi (28 °C), bacteria (32 °C) and actinomycetes (32 °C). The samples were cultured at a speed of 150 rpm for 7 days and then centrifuged for 15 min at 12,000 rpm to obtain the fermentation supernatant. The fermentation supernatant was filtered by 0.22 μm microfiltration membrane to obtain the fermentation filtrate. Inhibitory activity was tested by filter paper method [23]. A piece of round sterilized filter paper (d = 5 mm) with fermentation filtrate was placed at 20 mm away from the colony edge and cultured at 28 °C for 3 days to measure the width of the inhibition zone (mm). The sterile water was used as the blank control. Three replicates of each treatment were used, and the inhibition tests were performed three times. The mean of width of inhibition zone represented the inhibitory activity.

### Inhibition effect of fermentation filtrate on conidium germination of *A*.*alternata*

The spore germination test was accomplished using the hanging drop method [17]. Pathogen of JSFD was cultured on the PDA slope for a certain time. After full spore production, pathogen spores were washed down by a small amount of sterile water and then diluted by sterile water to approximately 40 spores per view under a microscope with 40 power magnification. The prepared spore suspension was mixed thoroughly with equal volume of fermentation filtrate, and 10 μL of the mixed suspension was dropped on a piece of concave slide. The spore suspension mixed with sterile water was used as the control group. The suspensions were cultured for 8 h at 28 °C and moisture-keeping condition. Spore morphology was observed in the middle view and five surrounding views under the microscope. The total spore quantity and germination quantity in each view were recorded. Germination was considered when the germ tube length of spores was larger than spore diameter.

The fermentation filtrate of screened endophyte St-zn-34 was mixed with equal volume of spore suspension of pathogen of JSFD into fermentation filtrates with different concentrations (10%, 20%, 30%, and 50%). The fermentation filtrate of the screened endophyte St-zn-34 mixed with sterile water was used as the control group. The filtrates were cultured for 8 h under humidity and 28 °C. The number of germinated spores and the total spore number were recorded. Three replicates of each treatment were used, and the experiments were performed three times. The germination rate and calibrated germination rate were calculated as follows: germination rate (%) = [germinated spores/totally checked spores] ×100%; and calibrated germination rate (%) = [germination rate of test group/ germination rate of control group] ×100%.

### Colonization capacity of St-zn-34 on jujube leaves

Resistance tag of strain St-zn-34 was conducted according to method of Glandorf et al [24]. Strain St-zn-34 was inoculated into a piece of NA plate with 5 μg/mL rifampicin and cultured at 28 °C. The new colony was inoculated into the same concentration of NA plate 48 h later and then transferred to the NA plate with 10 μg/mL rifampicin after once subculture, which was cultured at 28 °C. Next, the rifampicin concentration was added continuously until the mutant strains that could grow in the high-concentration (120 μg /mL) rifampicin and exhibited the same colonial morphology and antagonistic ability to pathogen of JSFD were screened. The colonization dynamics of St-zn-34 in jujube leaves were tested as follows. The sterile water for St-zn-34 was prepared into 1.5×10^9^ CFU/mL bacterial suspension. During the growth season of jujube, the bacterial suspension was coated to the jujube leaf surface using sterile cotton. The bacterial suspension treated with sterile water was used as the control group. Then, the strains were isolated from leaves every 5 days by the same method of endophyte isolation. The NA with 120 μg/mL rifampicin was used as the alternative plate culture medium. Three replicates of each treatment were included and the tests were conducted three times.

### Identification of the endophytic bacteria with biocontrol potential

#### Morphological observation

Bacterial morphology was observed using an environmental scanning electron microscope. The isolated and purified strains were selected into a shake flask with 10 mL of liquid culture medium, followed by 12 h of shake cultivation. In the next step, 5 mL of culture solution were collected and centrifuged for 5 min at 4000 rpm. The supernatant was discarded, but the sediment was obtained. The sediment was mixed fully with 4% glutaraldehyde for immobilization and then allowed to stand for 4 h at 4 °C. The mixture was centrifuged for 3 min at 4000 rpm. The supernatant was eliminated, and the precipitate was dehydrated in a gradient manner by 10%, 20%, 30%, 50%, 70%, and 90% ethyl alcohol. Every dehydration process took 10 min, followed by 3 min of centrifugation at 4000 rpm. Finally, the precipitate was dehydrated twice by 100% ethanol and then kept in a refrigerator at 4 °C. A small portion of the solution was dipped by a piece of brush pen to the objective table, followed by metal spraying and observation under microscope.

#### Physiological and biochemical characteristics

Isolated bacteria were tested according to conventional experimental methods (Fang, 1998), such as movement, dry resistance, temperature, Gram staining, endospore staining, V-P test, methyl red reaction, amylolysis, urease, gelatin liquefaction, salt tolerance, catalase, nitrate reduction, and oxidative fermentation. Characteristics were observed and recorded at 10 days (28 °C) according to *Bergey’s Manual of Determinative Bacteriology* [25] and *Manual for System Identification of Common Bacteria* [26].

#### DNA extraction, sequencing, sequence analysis, and establishment of the phylogenetic tree

The 16S rDNA genes of bacterial strains were amplified from the total DNA by the bacterial liquid PCR [27] using 27F (5′-AGAGTTTGATCCTGGCTCAG-3′) and 1492R (5′-TACTTGTTACGACTT-3′). The amplification reaction was conducted on the PCR instrument made by Bio-Rad, America. The amplification process included 30 cycles of 4 min of predegeneration at 95 °C, 30 s of degeneration at 94 °C, 30 s annealing at 56 °C, and 2 min of extension at 72 °C. The final solution was kept at 4 °C. Then, 5 μL of PCR products were collected, and the amplification results were tested by 1% agarose gel electrophoresis. The PCR amplification products were sent to Shanghai Sangon Biotechnology Co., Ltd for sequencing. The sequencing results were compared with the GenBank database through BLAST. Multiple sequence comparisons of homologous sequence and sequences of related standard strains were performed by Mega 6.0. The phylogenetic tree was constructed by Neighbor-Joining method to determine the taxonomic status of strains.

### Data analysis

Multiple comparisons of test data were implemented by Duncan’s new multiple range test using SPSS version 16.0 software.

## Results

### Observation and statistics on endophyte quantity in jujube

Table 1 shows that the endophyte quantity isolated from different organs of jujube trees varied. A total of 81 endophytic strains were isolated from different positions of the jujube tree. These strains include 37 fungi, 20 bacteria, and 24 actinomycetes.

**Table 1:** Number of endophytes isolated from different organs of Dongzao jujube.

Organs	Fungi	Bacteria	Actinomycetes
**Flower**	3	3	6
**Stem**	18	6	8
**Leaf**	9	6	6
**Fruit**	7	5	4
**Total**	37	20	24

### Dual culture results of different endophytes and *A*.*alternata*

Table 2 shows that among these 81 strains of jujube endophytes, 14 strains with antagonistic activities were selected in the dual culture. These strains have strong nutrient and spatial competition or antibiosis to the mycelial growth of *A*. *alternata*. The inhibition rates of St-zn-9, St-zn-12, St-zn-15, St-zn-21, St-zn-34, and St-zn-35 were higher than 50%. These strains were selected for the following screening.

**Table 2:** Antagonism of different endophytic strains against *A*. *alternata*.

Strains	Taxonomic classification	Sources	Colony diameter of the pathogen (mm)	Inhibition rate (%)
**St-zn-3**	Bacterium	Stem	24.07 ± 0.26	29.48 eD
**St-zn-4**	Bacterium	Flower	23.43 ± 0.12	31.35 deD
**St-zn-5**	Bacterium	Stem	27.33 ± 0.22	19.92 gF
**St-zn-9**	Fungus	Flower	7.20 ± 0.15	78.9 aA
**St-zn-12**	Actinomycete	Stem	17.37 ± 0.12	49.11 bB
**St-zn-15**	Fungus	Fruit	16.73 ± 0.12	50.98 bB
**St-zn-21**	Fungus	Flower	8.47 ± 0.30	75.18 aA
**St-zn-34**	Bacterium	Leaf	7.50 ± 0.06	78.03 aA
**St-zn-35**	Bacterium	Leaf	8.33 ± 0.13	75.59 aA
**St-zn-41**	Actinomycete	Fruit	25.93 ± 0.09	24.03 fE
**St-zn-42**	Actinomycete	Leaf	23.17 ± 0.23	32.11 dD
**St-zn-43**	Bacterium	Flower	27.27 ± 0.20	20.1 gF
**St-zn-45**	Bacterium	Leaf	29.40 ± 0.36	13.86 hG
**St-zn-46**	Fungus	Flower	19.67 ± 0.39	42.37 cC
**CK**			34.13 ± 0.09	0

Note: The data are the average of three replicates and presented as the means ± standard error. Different lowercase and capital letters in the same column indicated significant differences at 0.05 and 0.01 levels, respectively. CK: Control plates of *A*. *alternata* CN193 without endophytic strains.

### Inhibition effects of fermentation filtrates on *A*.*alternata*

**Fig 1:**
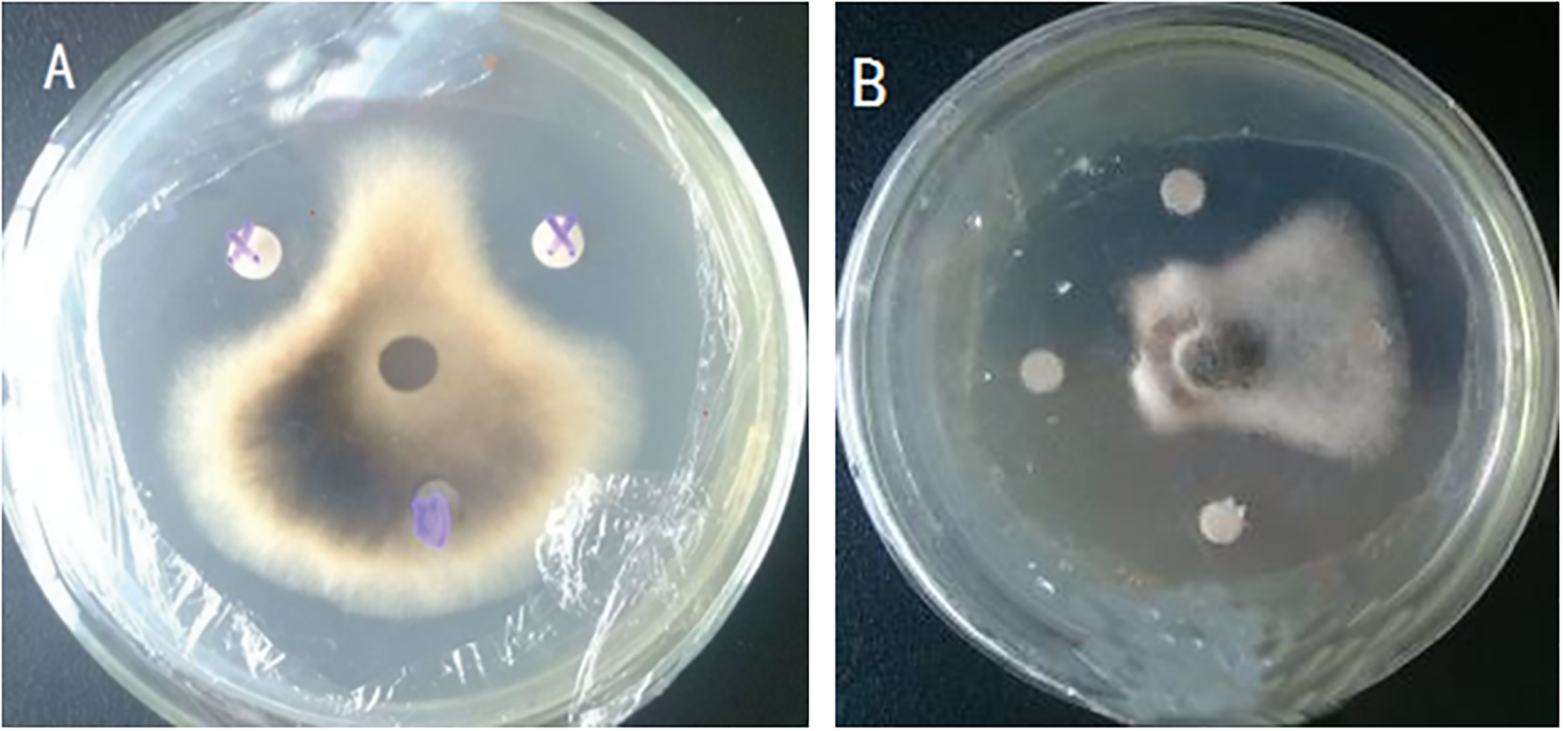
The inhibition effect of ferment of endophytic stains St-zn-9(A) and St-zn-34(B) against *A*. *alternate*.

The production abilities of antibiotics of preliminarily screened 14 strains were tested by fermental cultivation (Table 3). St-zn-3, St-zn-4, St-zn-5, and St-zn-15 could not produce any antibiotics, whereas the fermentation filtrate of the other strains, especially St-zn-9 and St-zn-34, could inhibit pathogen of JSFD. The inhibition zones of St-zn-9 and St-zn-34 reached 6.14±0.03 mm (Fig 1A) and 8.27±0.09 mm (Fig 1B), respectively. These two strains were further explored.

**Table 3:** Inhibition effects of the filtrates of 14 endophytes on *A*. *alternata*.

Strains	Width of inhibition zone (mm)
**St-zn-3**	0 hH
**St-zn-4**	0 hH
**St-zn-5**	0 hH
**St-zn-9**	6.14±0.03 bB
**St-zn-12**	3.37±0.07 dD
**St-zn-15**	0 hH
**St-zn-21**	3.97±0.09 cC
**St-zn-34**	8.27±0.09 aA
**St-zn-35**	3.10±0.12 eE
**St-zn-41**	3.97±0.07 cC
**St-zn-42**	3.00±0.12 eE
**St-zn-43**	1.00±0.06 gG
**St-zn-45**	2.03±0.15 fF
**St-zn-46**	2.13±0.09 fF
**CK**	0 hH

Note: The data are the average of three replicates and presented as the means±standard error. Different lowercase and capital letters in the same column indicated significant differences at 0.05 and 0.01 levels, respectively. CK: Sterile water.

### Effect of fermentation filtrate on spore germination of *A*.*alternata*

**Fig 2:**
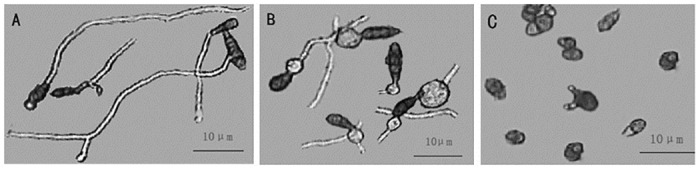
Microscopic observation on the suppression effects of strain St-zn-34 filtrate on the spore germination of *A*. *alternata* (bar = 10 μm). Spore germination in (A) sterile distilled water, (B) filtrate of strain St-zn-9, and (C) filtrate of strain St-zn-34.

**Fig 3:**
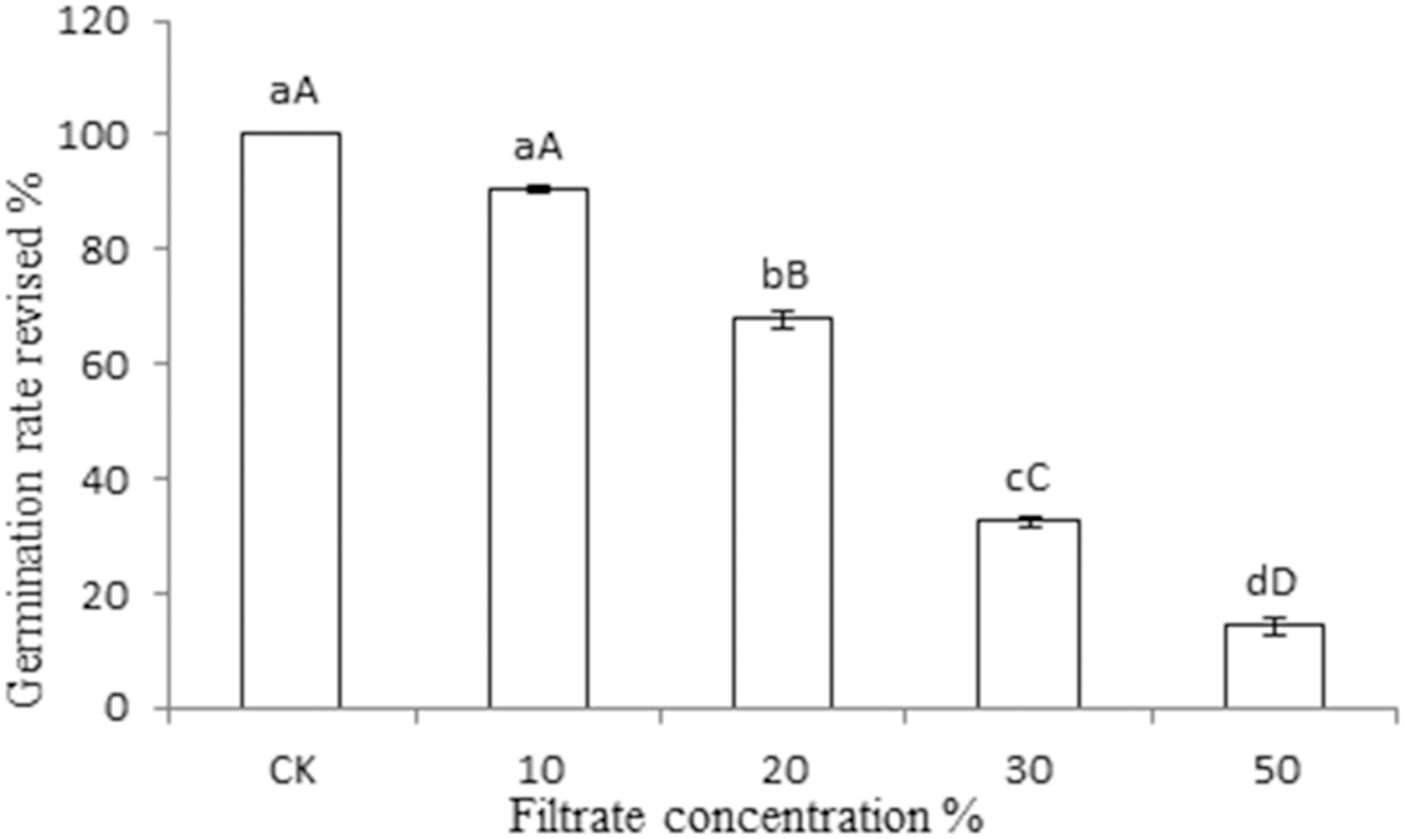
Effects of different concentrations of filtrate of endophytic strain St-zn-34 on the spore germination rate of *A*. *alternate*. Note: Data are the average of three replicates and presented as the means±standard error. Different lowercase and capital letters in the same column indicate significant differences at 0.05 and 0.01 levels, respectively.

The spore germination inhibition test was performed using St-zn-9 and St-zn-34, and the results are shown in Fig 2. Pathogen of JSFD could germinate normally in sterile water, showing smooth and slim germ tube (Fig 2A) and complete germination of all spores. In the fermentation filtrate of St-zn-9, a big vesicle was produced before spore germination, and then germ tube was produced (Fig 2B). All spores germinated. No significant difference was found in the germination rates compared with the control. However, spores germinated rarely or just begin to germinate in the fermentation filtrate of St-zn-34, which showed short germ tube or 3 germ tubes at one end of several spores. This result reflects the strong inhibition of spore germination in St-zn-34 (Fig 2C). In Fig 3, the inhibiting effects of the different concentrations of fermentation filtrate of St-zn-34 on spore germination in pathogen of JSFD varied. By combining data of Tables 2 and 3, St-zn-34 was chosen as the biocontrol strain for subsequent tests.

### Test of colonization capacity

**Fig 4:**
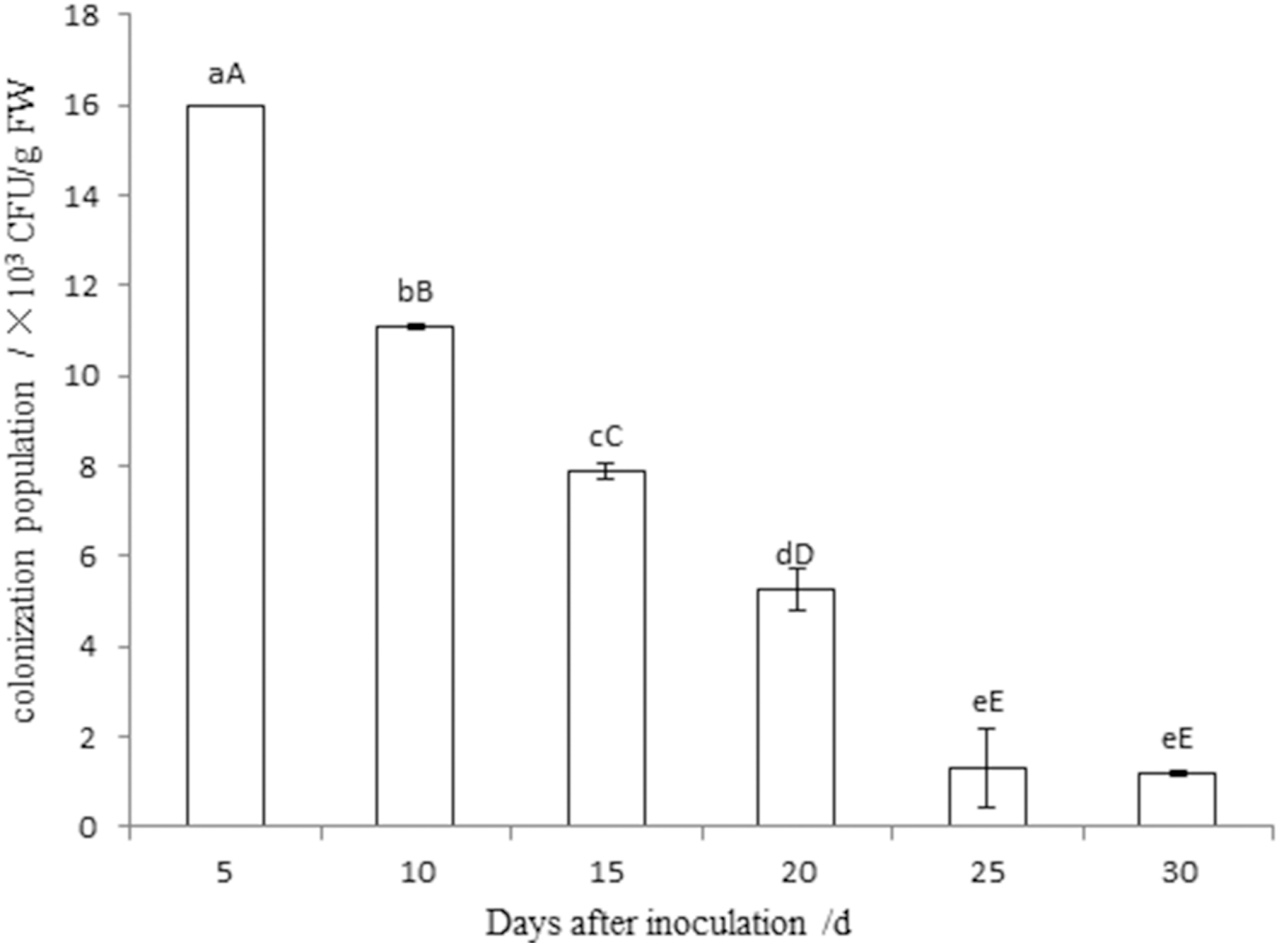
Population dynamics of strain St-zn-34^r^ in the leaves of Dongzao jujube after inoculation. Note: The data are the average of three replicates and presented as the means±standard error. Different lowercase and capital letters in the same column indicated significant differences at 0.05 and 0.01 levels, respectively.

One strain of rifampicin-resistant mutant strain St-zn-34^r^ was acquired through gradual induction of antibiotics. Contrary to the original strain, the mutant strain could grow normally on the NA plate with 120 μg/mL rifampicin. The strains had completely consistent physiological and biochemical characteristics and colony morphology on NA plate without antibiotics. Fig 4 shows that the colonization quantity of St-zn-34^r^ on leaves decreased gradually with time, but the strains persisted and reached 1.2×10^3^ CFU/g FW. These results show the strong colonization of the pathogen on jujube leaves. Nevertheless, no mutant strains were isolated from the leaves in the control group.

### Identification of endophytic bacteria St-zn-34

#### Morphological features

**Fig 5:**
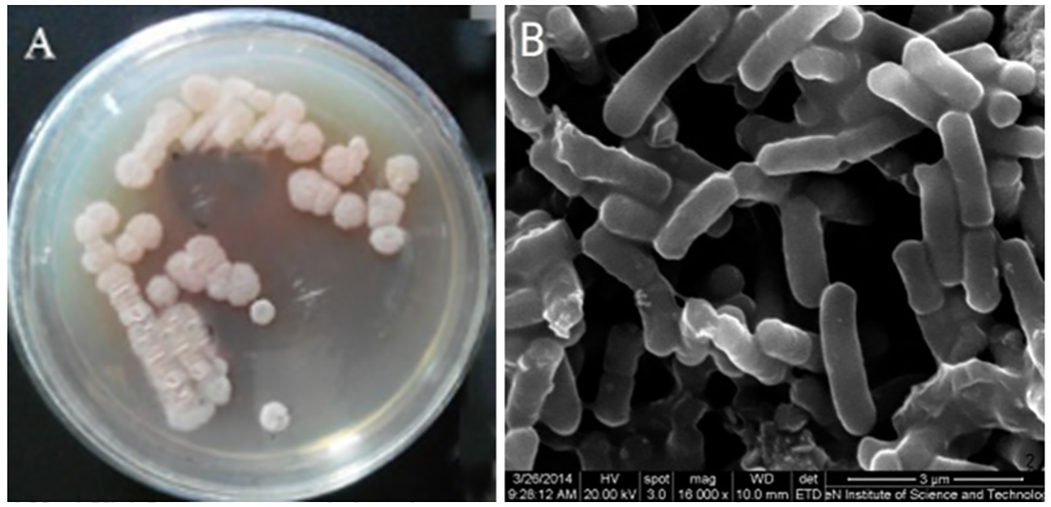
Culture characteristic in PDA (A) and microscopic observation under environment scanning electron microscope (B) of St-zn-34 (bar = 3 μm).

The strain produced different pigments on the various culture media. The strain produced red pigment 2 days after inoculation on the PDA medium but milk white pigment on the beef extract peptone medium. The colonies showed similar sizes, round shapes, unsmooth edges, and milk white surface bulges. Moreover, the colonies had smooth surface, wrinkle edges, and 0.4–0.6 mm in diameter (Fig 5A). Bacteria were observed as rods under the microscope, with size ranging between 2.01–2.56 μm×0.64–0.71 μm (Fig 5B).

#### Physiological and biochemical characteristics

The physiological and biochemical characteristics of St-zn-34 are listed in Table 4. The strain showed positive Gram staining, motility, facultative anaerobes, normal growth at initial pH of 5–9 and 50°C, and utilization of citrate but not propionate. The strain tested negative in methyl red reaction, hydrolysis of starch, and gelatin, and in urease. The strain could grow well at 3% NaCl but showed inhibited growth at over 5% NaCl. The strain also showed positive catalase, positive nitrate, and positive V-P tests. According to the studies of Buchanan & Gibbons [25], Dong & Cai [26] and Liu et al. [28], St-zn-34 showed the closest physiological and biochemical characteristics with *Bacillus subtilis*, except for some difference in salt resistance.

**Table 4:** Physiological and biochemical characteristics of St-zn-34.

Tested items	St-zn-34	*Bacillus subtilis*
**Gram staining**	+	+
**Motility**	+	+
**Endospore**	+	+
**Oxidative fermentation**	Facultative anaerobic	Aerobiotic or facultative anaerobic
**Initial pH 5–9**	+	+
**Growth at 50°C**	+	+
**Citrate utilization**	+	+
**Propionate utilization**	−	−
**Methyl red test**	−	−
**Starch hydrolysis**	+	+
**Urease**	−	−
**Gelatin liquefaction test**	+	+
**Growth in 3% NaCl**	+	+
**Growth in 5% NaCl**	−	+
**Growth in 7% NaCl**	−	+
**Catalase test**	+	+
**Nitrate reduction**	+	+
**V-P test**	+	+

Note: “+” indicates positive reaction; “−”indicates negative reaction

#### Analysis of 16S rDNA sequences

**Fig 6:**
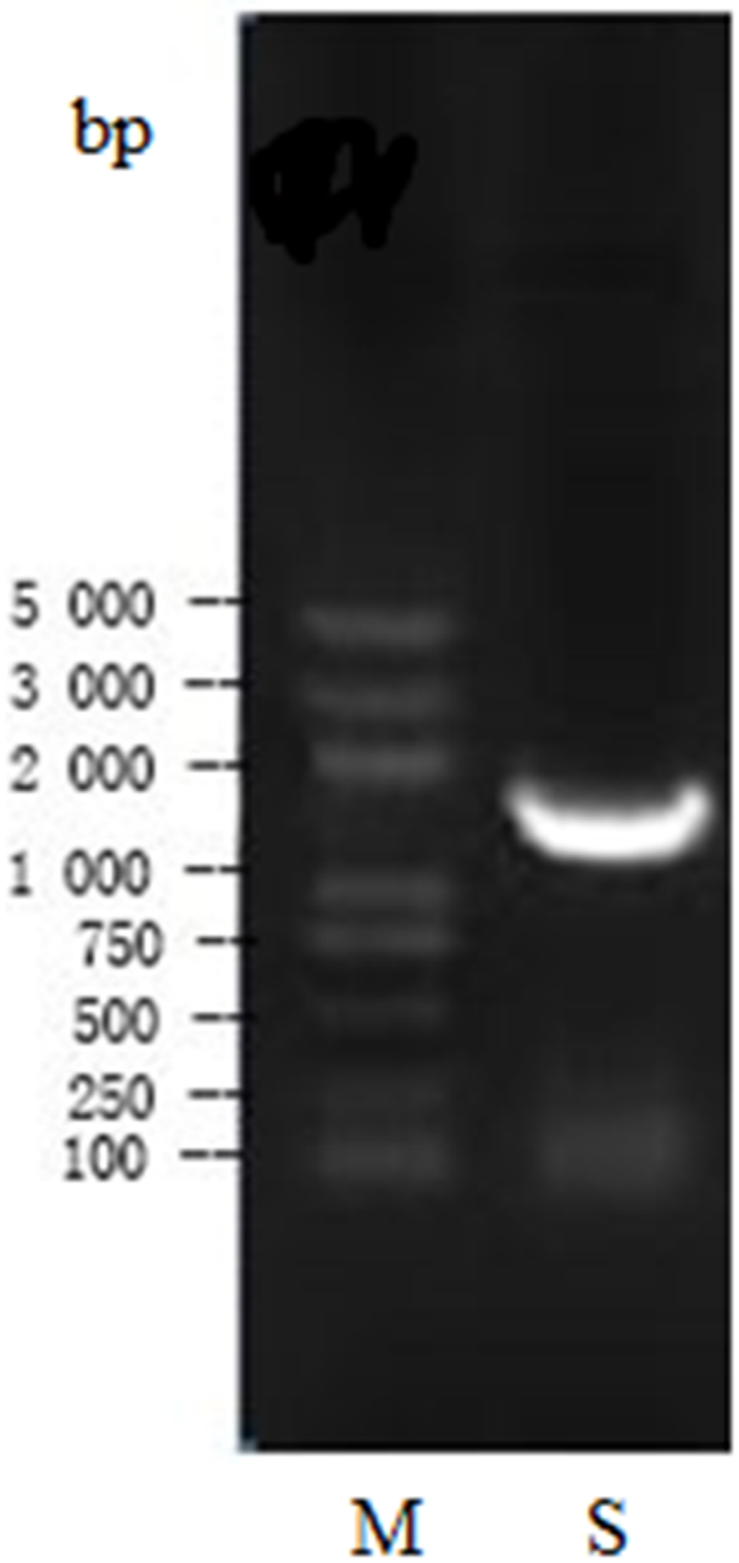
Electrophoretogram of PCR amplicons of 16S rDNA gene. M: Marker; S: Sample.

**Fig 7:**
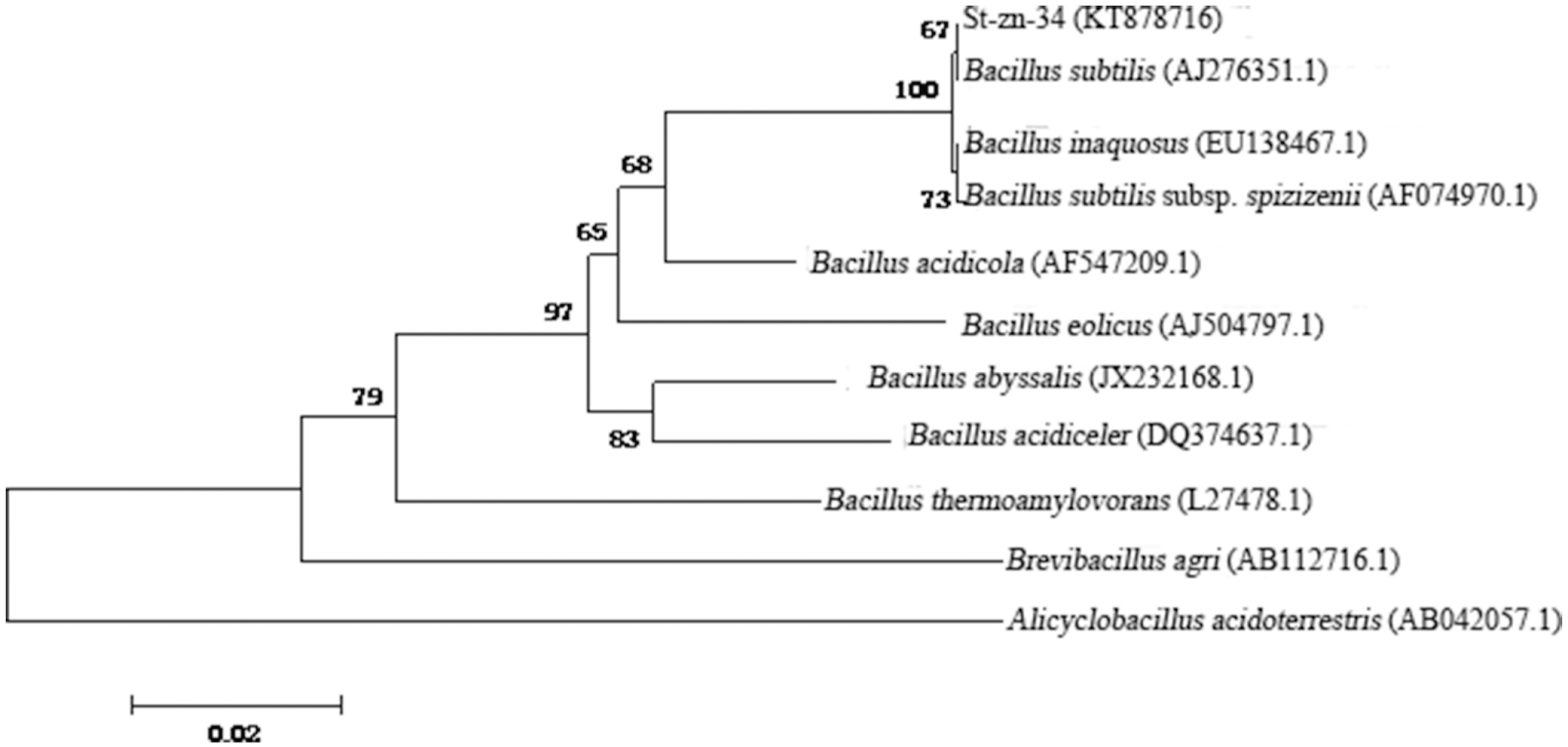
Phylogenetic tree of St-zn-34 based on 16S rDNA sequence.

The 16S rDNA product of St-zn-34 after specific PCR amplification showed a clear belt after electrophoresis staining, without other belts. The target sequence size was approximately 1400 bp (Fig 6). The sequence of the 16S rDNA of St-zn-34 strain (accession number KT878716) was submitted to GenBank and it showed over 98% homology with *Bacillus* strains. Thus, St-zn-34 may belong to *Bacillus*. In this paper, 16S rDNA sequences of type strains of nine *Bacillus* families were chosen from the GenBank as the ingroup, and 16S rDNA sequence of *Alicyclobacillus acidoterrestris* was used as the outgroup. The phylogenetic tree was constructed after multiple comparisons based on Mega 6.0 (Fig 7). The results demonstrated that St-zn-34 has shorter genetic distance with *B*. *subtilis* compared with other *Bacillus* strains. The two strains are on the same branch. Thus, St-zn-34 was identified *B*. *subtilis* on the basis of morphological, physiological, and biochemical characteristics as well as the analysis of 16S rDNA sequence.

## Discussion

A total of 81 endophytic strains were isolated from different organs of a healthy winter jujube. These strains included 37 fungi, 20 bacteria, and 24 actinomycetes strains. Among these strains, 14 endophyte strains with certain spatial and nutritional competition were screened. Two strains (St-zn-9 and St-zn-34) with strong inhibition effects were selected by filter paper method. The inhibition zones of St-zn-9 and St-zn-34 were 6.14 ± 0.04 mm and 8.27 ± 0.11 mm, respectively. Effects of fermentation products on spore germination of the causal agent of JSFD were tested by the hanging drop method. St-zn-34 could significantly inhibit spore germination. Therefore, St-zn-34 was screened as the biocontrol bacteria. St-zn-34 was identified as *B*. *subtilis* based on the morphological characteristics, physiological and biochemical characteristics and analysis of 16S rDNA sequence.

The name of JSFD was confusion and the pathogen was controversial. However, many scholars have proved recently that *A*. *alternata* was the pathogen or one of the pathogens of JSFD [3,29–32]. Therefore, *A*. *alternata* was chosen as the target pathogen of biocontrol strain of jujube endophytes. Culturable endophytes were isolated from different organs of jujube trees by conventional method. However, this process may fail to identify some non-culturable strains and some strains that grow slowly in the traditional culture medium. Population distribution patterns gained from non-culture and culture method differed significantly. The selected biocontrol strain St-zn-34 was identified as *B*. *subtilis* and isolated from jujube leaves. Xu et al. [3] successfully isolated *B*. *subtilis* from jujube fruits, indicating its universal distribution in jujube trees.

Two selected endophytes strains (St-zn-09 and St-zn-34) showed good spatial and nutritional competition and strong antibiotics production. In this study, St-zn-09 was preliminarily identified as *Trichoderma* according to colonial morphology. This strain influenced the spore germination of *A*. *alternata* but differed significantly from the conidium germination of the control group. Conidia always produce a vesicle before each germination process, similar to the finding of Zhang et al. [33]. This process may be due to the fact that St-zn-09 could break spore cell walls of *A*. *alternata* and cause outburst of protoplasm, though St-zn-09 did not affect spore germination of *A*. *alternata*. St-zn-34 inhibited the conidium germination of *A*. *alternata*, which conforms to the finding of Gu et al. [34]. The transmission of pathogen of JSFD is determined by its spores, so the strong inhibiting effect of the fermentation products of St-zn-34 on spore germination will directly affect the transmission ability of pathogen in the field. The antibiotics ability of biological strains is an important evaluation index of biocontrol effect. *B*. *subtilis* can produce *iturins*, *fengycins*, *mycosubtilins*, and many antibiotic substances. The antibiotic substance produced by St-zn-34 fermentation has been proved to exhibit wide antibacterial spectra and stable convective heat transfer, conforming to the characteristics of bacterial inhibition and heat tolerance of lipopeptide antibiotics. The proteinase K could reduce its bacterial inhibition, which reflects that it had properties of protein. Therefore, proteinase K should be a bacterial-inhibiting protein substance [35]. However, the biocontrol agent had the weakness of poor control effect and short duration in field application, which was related to the weak colonization capacity of the biocontrol agent in the host plants. The strong colonization capacity of the endophyte in the host could effectively reduce the accumulation of pathogen, and thus control disease [36]. Therefore, studies on the colonization of St-zn-34 in jujube trees will provide a theoretical basis for the biocontrol of JSFD. The endophytes screened in this paper exhibited strong colonization capacity and can maintain a long time duration in jujube leaves, but whether the strain could be colonized in other parts of jujube trees or it could spread from jujube leaves to other parts of jujube trees need further studies.

The salt concentration was also an important factor for St-zn-34. St-zn-34 grew well on 3% NaCl but not in solution with over 5% NaCl. This result is not consistent with the finding by Wang et al. [37] which might be related to the poor salt resistance of St-zn-34 as an endophyte. However, the main effect of an endophyte is to increase significantly the salt resistance of host after being colonized in plants [38]. Moreover, *B*. *subtilis* at low salt concentration could promote plant growth and would not significantly affect plant growth at high salt concentration [39]. Thus, the salt-stress physiology of this endophyte requires further investigation in the future.

## Supporting information

S1 Fig Streak culture of endophytic *Bacillus subtilis* strain St-zn-34 on potato dextrose agar plate. (TIF)

S2 Fig Confront co-culture of *Bacillus subtilis* strain St-zn-34 and *Alternaria alternata* strain CN193, the pathogen of jujube shrunken-fruit disease. (TIF)

S3 Fig Inhibition effects of fermentation filtrates of strain St-zn-34 on *A*.*alternata* strain CN193. (TIF)

S4 Fig Microscopic observation of strain St-zn-34 under environment scanning electron microscope. (TIF)

S5 Fig 16S rDNA gene sequence of strain St-zn-34. (TIF)
