# Supplementary figures and images for: Identification of endophytes with biocontrol potential from Ziziphus jujuba and its inhibition effects on Alternaria alternata, the pathogen of jujube shrunken-fruit disease
Source: PLoS One. 2018 Jun 26;13(6):e0199466. doi: 10.1371/journal.pone.0199466 (PMC6019103; doi:10.1371/journal.pone.0199466)

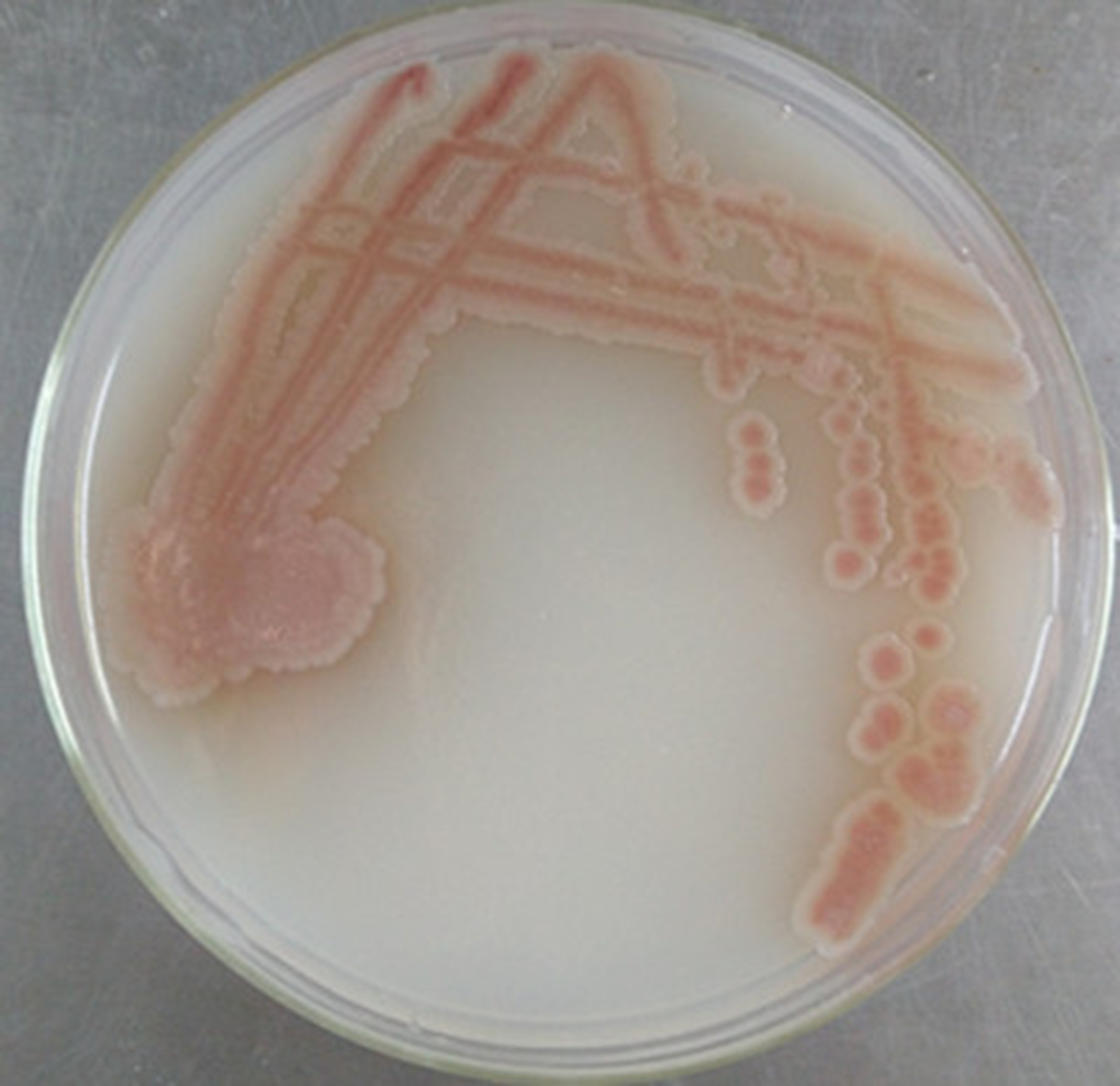

Supplement: S1 Fig — (TIF) [file pone.0199466.s001.tif]

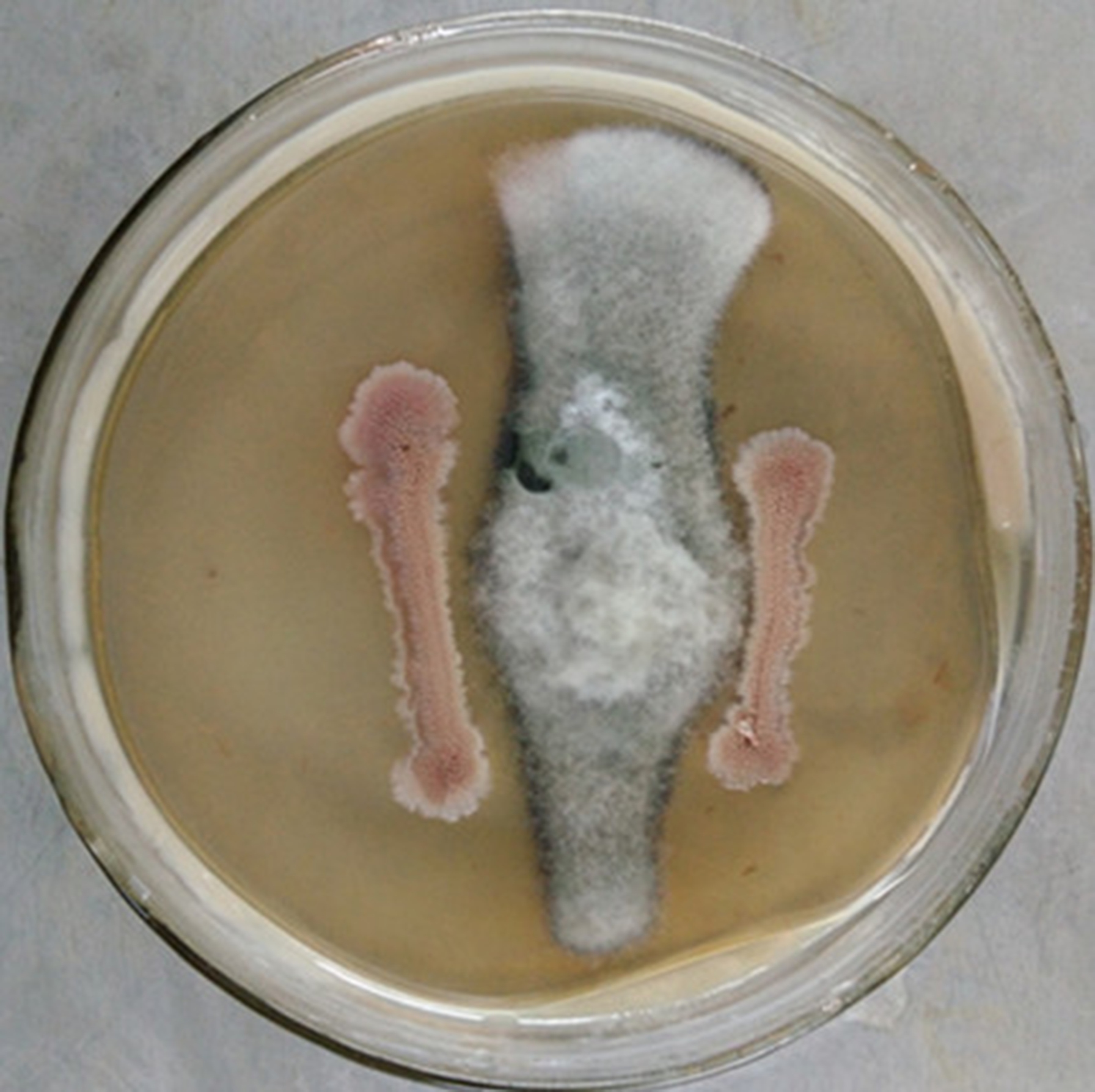

Supplement: S2 Fig — (TIF) [file pone.0199466.s002.tif]

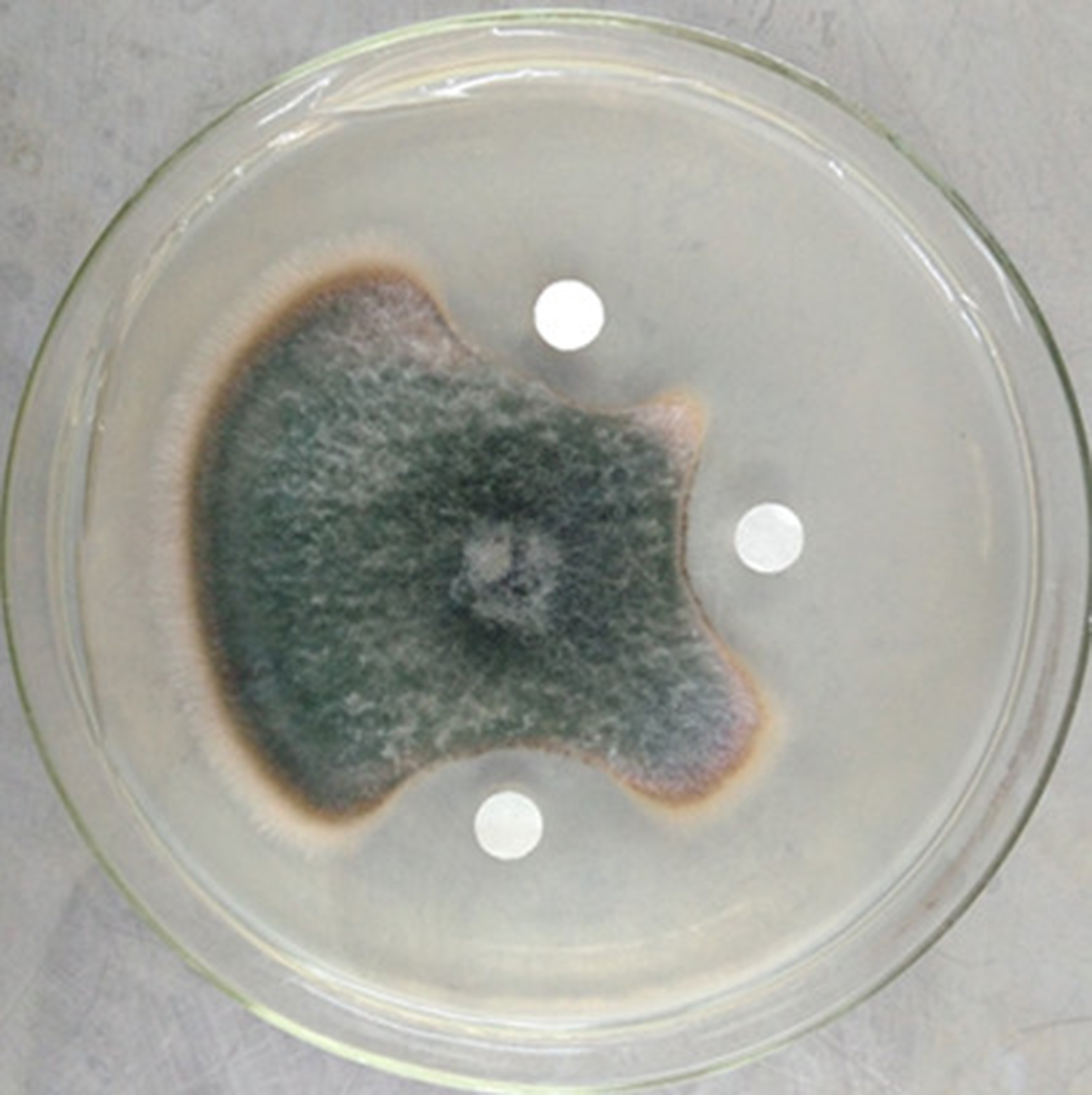

Supplement: S3 Fig — (TIF) [file pone.0199466.s003.tif]

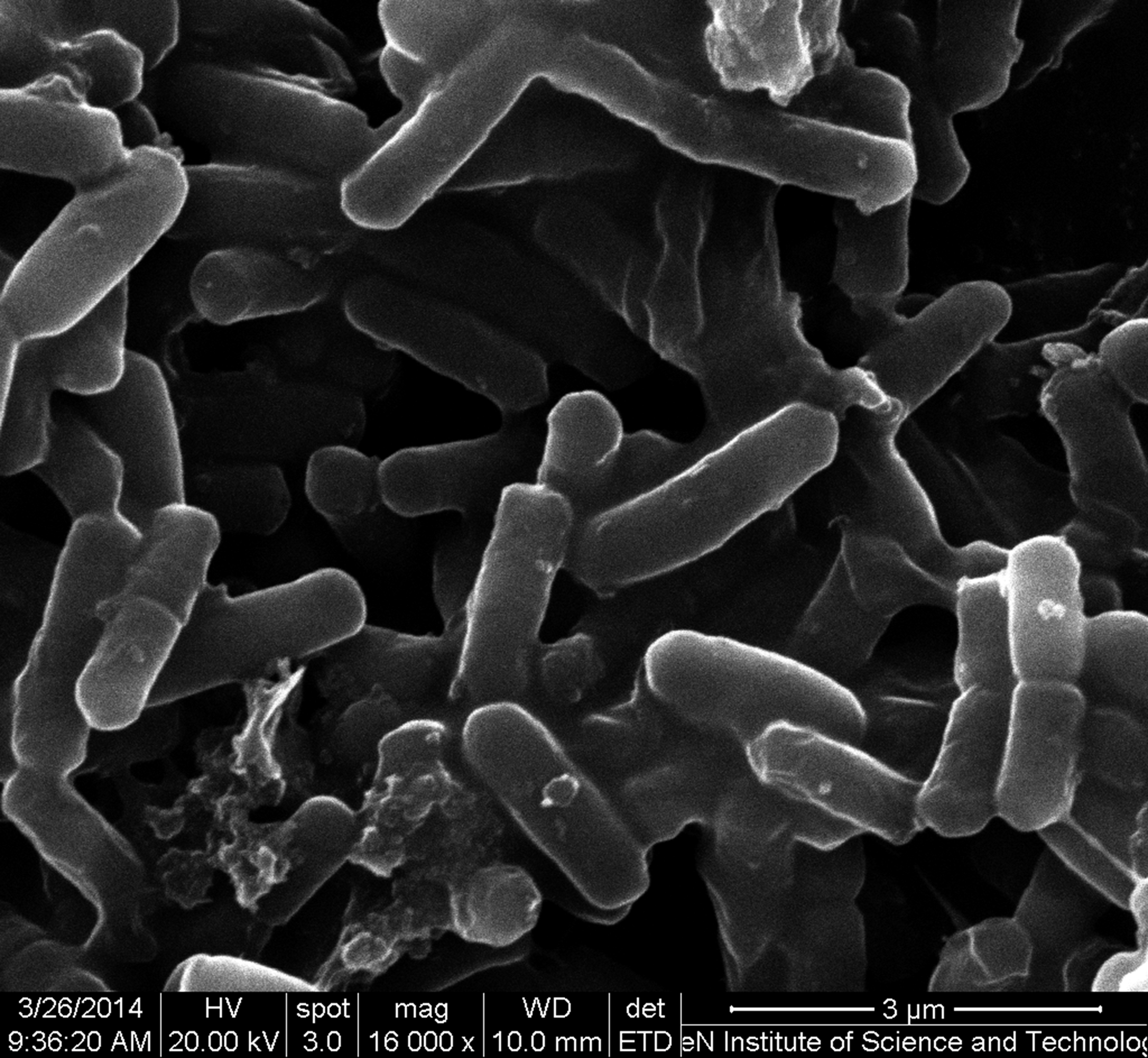

Supplement: S4 Fig — (TIF) [file pone.0199466.s004.tif]

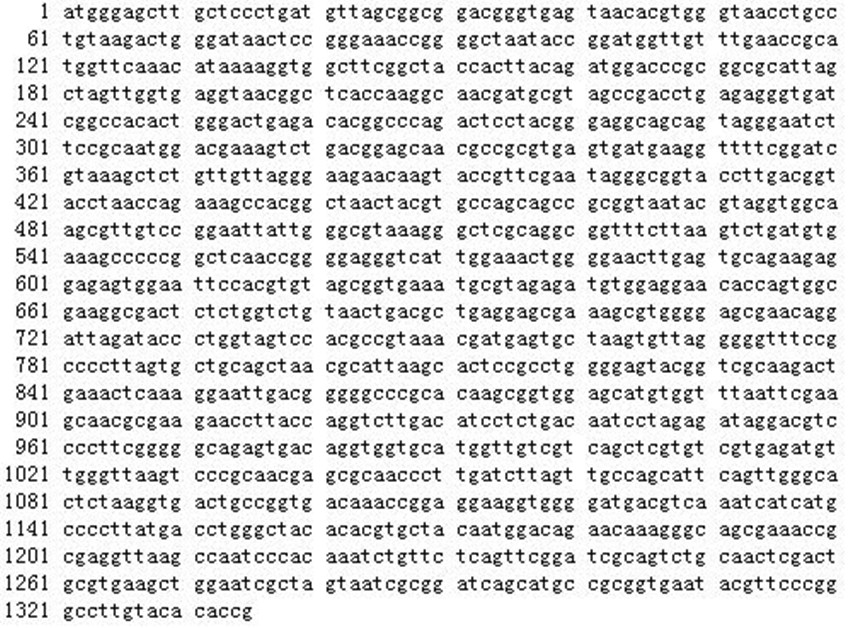

Supplement: S5 Fig — (TIF) [file pone.0199466.s005.tif]
